# Synergistic Effects of Fiber Inclination, Geometry, and Thermal Treatment on Fe-SMA Fiber Pull-Out Resistance in High-Performance Concrete

**DOI:** 10.3390/ma19081668

**Published:** 2026-04-21

**Authors:** Jan Białasik, Wojciech Podraza, Dominika Samulczyk, Alireza Tabrizikahou

**Affiliations:** Institute of Building Engineering, Poznan University of Technology, Piotrowo 5, 60-965 Poznan, Poland; jan.bialasik@put.poznan.pl (J.B.); wojciech.podraza@student.put.poznan.pl (W.P.); dominika.samulczyk@student.put.poznan.pl (D.S.)

**Keywords:** iron-based shape memory alloy (Fe-SMA) fibers, pull-out behavior, fiber inclination angle, fiber geometry, thermal treatment, concrete–fiber bond

## Abstract

Iron-based shape memory alloy (Fe-SMA) fibers can enhance cementitious composites through both crack bridging and thermally activated recovery stresses. Since fiber pull-out governs load transfer at the micro scale, understanding the combined effects of fiber geometry, inclination, and thermal treatment is essential. This study experimentally investigated the pull-out behavior of hooked-end Fe-SMA fibers embedded in high-performance concrete (HPC). A total of 54 ASTM C307-type briquette specimens were tested using single-hook (3D) and double-hook (4D) fibers at inclination angles of 60°, 75°, and 90° under ambient, 100 °C, and 200 °C conditions. Additional flexural, compressive, and direct tensile tests were conducted on plain HPC exposed to the same thermal regime. At ambient temperature, 4D fibers showed 50–70% higher peak pull-out forces than 3D fibers. Heating to 100 °C further increased pull-out resistance by about 6–17%, and the 4D-60-100 configuration achieved the highest performance. In contrast, exposure to 200 °C reduced pull-out resistance by about 5–12% below ambient values. Overall, a 60° inclination generally provided a better response, while 90° produced the lowest. The results confirm that moderate thermal activation combined with double-hook geometry is the most effective strategy for maximizing Fe-SMA fiber–matrix load transfer in HPC.

## 1. Introduction

Fiber-reinforced concrete (FRC) has become a popular material in structural engineering because short discrete fibers can improve crack control, post-cracking load transfer, toughness, impact resistance, and durability. However, these benefits do not depend only on fiber volume fraction or tensile strength; they are governed to a large extent by the interaction between the fiber and the surrounding cementitious matrix [1,2]. In practice, the pull-out resistance of a fiber is one of the key micromechanical parameters controlling crack bridging, energy dissipation, and the flexural and tensile response of the composite. Accordingly, understanding the bond and pull-out behavior of fibers is essential for the rational design of high-performance cementitious composites [3].

For conventional steel-fiber-reinforced concrete, it is well established that pull-out behavior is controlled by a combination of adhesion, friction, and mechanical anchorage, especially when hooked-end fibers are used [4]. The geometry of the fiber end, the strength of the surrounding matrix, and the orientation of the fiber relative to the loading direction all influence the resulting pull-out load–slip response [5]. Previous studies on steel fibers showed that fiber inclination can increase bond resistance through a snubbing effect, but excessive inclination may also cause matrix spalling, fiber rupture, or a reduction in effective anchorage length [6]. More recent work on 3D, 4D, and 5D hooked-end steel fibers confirmed that fiber orientation and hook geometry remain decisive variables, while also indicating that the response becomes increasingly complex when multiple anchorage bends and higher-strength matrices are involved [7].

**Figure 1:**
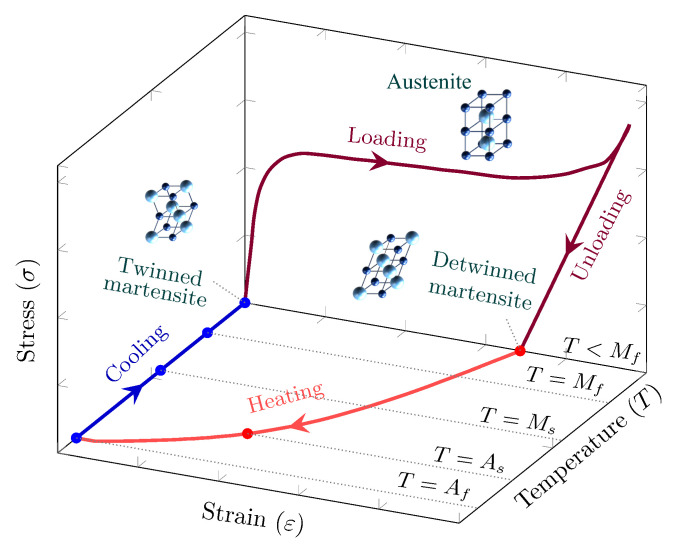
Shape memory effect and martensitic phase transformation due to thermal treatment in SMA [16].

In parallel with these developments, shape memory alloy (SMA) fibers have attracted growing interest because they can contribute functionalities beyond those of passive steel fibers. Owing to shape memory effect (see Figure 1) and superelasticity, SMA fibers can provide crack-closing, self-centering, re-centering after unloading, and thermally activated recovery stresses [8,9,10]. Most of the early work in this area was performed with NiTi-based fibers [11]. Studies on NiTi fibers demonstrated that end shape, cold drawing, and heat treatment strongly affect pull-out resistance, while beam and mortar investigations showed that SMA fibers can improve flexural performance and facilitate crack closure or recovery after damage [12,13,14,15]. These studies clearly established the promise of SMA-FRC, but they also highlighted that the effectiveness of SMA fibers is inseparable from their bond behavior at the fiber–matrix interface.

Despite the success of NiTi systems, their high material cost remains a serious limitation for widespread use in civil engineering. For this reason, iron-based shape memory alloys (Fe-SMAs) have emerged as an attractive alternative [17,18]. Fe-SMAs offer lower cost, good workability, relatively high stiffness, and the ability to generate recovery stress after thermal activation, which makes them particularly suitable for large-scale structural applications [19]. Over the past decade, most Fe-SMA research in construction has focused on bars, strips, and externally or near-surface mounted strengthening systems [20]. These studies demonstrated that Fe-SMA elements can successfully prestress or strengthen reinforced concrete members and have helped move the material from laboratory validation toward practical infrastructure applications. Nevertheless, these configurations differ fundamentally from short randomly distributed fibers, where the governing mechanism is not only recovery stress generation, but also the ability of each individual fiber to transfer that stress to the matrix through adequate anchorage and pull-out resistance.

For short Fe-SMA fibers, the importance of pull-out resistance is even greater because thermal activation may create two competing effects. On one hand, heating activates the reverse martensitic transformation and can generate recovery stresses that improve crack bridging or prestressing action [21]. On the other hand, heating may weaken the surrounding matrix or deteriorate interfacial bond conditions, thereby reducing the effective anchorage of the fiber. Recent work on Fe-SMA fibers has already shown that this balance is critical. A recent experimental study on Fe-SMA fiber pull-out reported that end-hooked fibers exhibited roughly twice the pull-out resistance of straight fibers, while elevated temperature substantially reduced the pull-out resistance of end-hooked fibers; the same study also showed that higher matrix strength increased pull-out resistance and that loading-rate effects were comparatively small [22]. At the composite level, a subsequent current study demonstrated that thermally activated short Fe-SMA fibers can prestress concrete and enhance flexural strength, but it also indicated that higher activation temperatures may become less beneficial because of degradation in fiber–matrix interaction [23]. Together, these findings show that any structural use of short Fe-SMA fibers must be supported by a deeper micromechanical understanding of how anchorage geometry and fiber orientation behave under thermal exposure.

**Figure 2:**
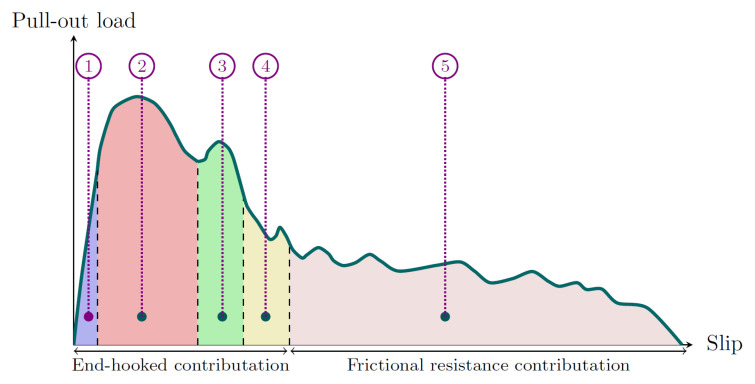
Schematic load–slip response of a hooked-end fiber during pull-out, showing the five characteristic stages and the relative contributions of end-hooked anchorage and frictional resistance.

**Figure 3:**
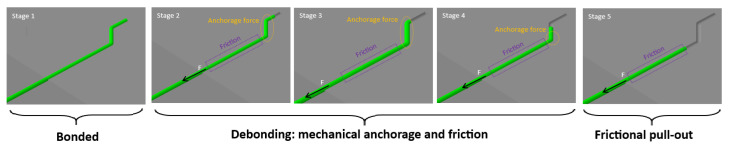
Five stages of the hooked-end fiber pull-out process: Stage 1—fully bonded; Stages 2–4—debonding with mechanical anchorage and friction; Stage 5—frictional pull-out.

The pull-out behavior of hooked-end fibers from a cementitious matrix proceeds through five distinct stages, as illustrated in Figure 2 and Figure 3.

In Stage 1, the fiber is fully bonded to the surrounding matrix and no slip occurs. The load is transferred entirely through chemical adhesion at the fiber–matrix interface, and the response is essentially linear elastic. As the applied load increases, debonding initiates at the loaded end and propagates along the fiber length. In Stage 2, the adhesive bond is progressively broken and the hooked end begins to engage the matrix, generating a mechanical anchorage force in addition to the frictional resistance along the debonded length. This stage corresponds to the first peak on the load–slip curve, representing the maximum combined contribution of adhesion, friction, and mechanical anchorage. In Stage 3, the hook undergoes plastic deformation as it is pulled through the matrix. The anchorage force reaches its second local maximum as the hook straightens and bears against the surrounding concrete, while frictional resistance continues to act along the entire debonded length. Stage 4 marks the completion of hook straightening. The anchorage force drops sharply as the deformed end clears the matrix, producing a local minimum on the load–slip curve. Frictional resistance remains the dominant load-carrying mechanism at this point. In Stage 5, the hook has been fully straightened and pulled out of its original cavity. The fiber slides freely through the matrix channel, and the response is governed entirely by frictional resistance, which decreases gradually as the embedded length reduces until complete pull-out occurs.

The inclination angle of the fiber with respect to the loading direction has a significant influence on the pull-out response, as demonstrated in several studies on hooked-end steel fibers. Yao and Leung [24] showed that for small inclination angles (0–10° from perpendicular), the peak pull-out force remains similar, whereas larger angles (20–30°) may induce matrix spalling at the fiber exit point and reduce peak resistance through shortening of the effective anchorage length. Also, they demonstrated that increasing inclination angle raises the transverse force pressing the fiber against the matrix at the exit point, thereby enhancing local friction through the snubbing effect; However, for large angles and stiff fibers, the resulting bending and contact stresses may initiate matrix cracking or premature fiber failure, suggesting the existence of an optimal inclination range rather than a monotonic increase in pull-out resistance. Zhang et al. [25] confirmed through acoustic emission and microscopic observation that greater inclination generally increases bond stress due to a stronger snubbing effect, while simultaneously intensifying local matrix damage at the exit zone; at excessively large angles, the reduction in effective anchorage length and matrix degradation may lower post-peak resistance and total pull-out energy despite a higher initial peak force.

The total pull-out resistance of a hooked-end fiber embedded at an inclination angle is therefore governed by the combined interplay of chemical adhesion, mechanical anchorage provided by the hook geometry, interfacial friction, and the snubbing effect at the fiber exit point. The relative contribution of each mechanism depends on the fiber geometry, inclination angle, and the thermal history of the specimen.

Although the recent literature has substantially advanced the understanding of SMA fibers in cementitious composites, the available knowledge remains incomplete for the specific problem addressed in the present work. NiTi-based studies have investigated pull-out behavior, end shape, and heat-treatment effects, while Fe-SMA studies have so far concentrated mainly on basic pull-out parameters, recovery behavior, or composite-scale flexural prestressing. Based on the published sources identified here, no prior study appears to have systematically examined the combined influence of Fe-SMA fiber inclination angle, hooked-end geometry, and thermal treatment on single-fiber pull-out resistance in high-performance concrete (HPC). This unresolved coupling is highly relevant because, in real composites, fibers are randomly oriented and therefore rarely aligned with the principal crack-opening direction; at the same time, changes in hook geometry and thermal history may alter the balance among adhesion, friction, snubbing, and local matrix damage.

Based on the current state of research, the main gaps in knowledge can be summarized as follows:

- The pull-out behavior of Fe-SMA fibers embedded in HPC under different inclination angles has not been adequately quantified, despite the fact that fiber orientation is a key variable in real randomly distributed composites.
- The influence of more advanced hooked-end geometries on Fe-SMA fiber anchorage remains insufficiently understood, particularly when compared with the more mature steel-fiber literature and the mostly NiTi-based SMA-fiber studies.
- The coupled effect of thermal treatment and fiber geometry on Fe-SMA pull-out is still unclear, even though thermal activation is indispensable for mobilizing the shape memory effect and may simultaneously damage the matrix or the bond interface.
- There is still limited experimental guidance for selecting the most effective combination of Fe-SMA fiber geometry, orientation, and activation temperature for future thermally activated Fe-SMA cementitious composites.

Accordingly, this study aims to address these gaps by:

- Experimentally evaluating the pull-out resistance of Fe-SMA fibers embedded in HPC while varying three governing parameters simultaneously: fiber end geometry, inclination angle, and thermal treatment.
- Comparing single-hook (3D) and double-hook (4D) Fe-SMA fibers at inclination angles of 60°, 75°, and 90° under ambient, 100 °C, and 200 °C conditions in order to clarify their individual and combined effects on the pull-out response.
- Characterizing the companion mechanical properties of plain HPC under the same thermal conditions so that the observed pull-out behavior can be interpreted in relation to changes in the surrounding matrix.
- Identifying the most favorable combination of geometry, inclination, and thermal exposure for maximizing fiber–matrix load transfer, thereby providing a micromechanical basis for the future design of Fe-SMA fiber-reinforced HPC with thermally activated functionality.

The present study therefore investigates the synergistic effects of fiber inclination, end-hook geometry, and thermal treatment on the pull-out resistance of Fe-SMA fibers embedded in HPC. To this end, 54 briquette specimens were tested using ASTM C307-type geometry, covering two fiber geometries, three inclination angles, and three thermal conditions, while additional companion specimens were used to determine the thermal-dependent properties of the plain HPC matrix. The results are intended to provide both fundamental insight into Fe-SMA fiber anchorage mechanisms and practical guidance for the future development of thermally activated Fe-SMA cementitious composites. The remainder of the paper presents the materials and experimental methods (Section 2), followed by the test results and discussion (Section 3), and finally the main conclusions (Section 4).

## 2. Experimental Program

This section presents the materials, specimen preparation procedures, and test methods employed to investigate the pull-out resistance of Fe-SMA fibers embedded in HPC under the combined effects of fiber geometry, inclination angle, and thermal treatment, as well as the mechanical properties of plain HPC.

### 2.1. Materials

The experimental program employed HPC prepared with a water-to-binder ratio of 0.31. The aggregate skeleton consisted of two quartz sand fractions—fine (0–0.5 mm) and coarse (0.5–1.0 mm)—while the binder system combined CEM II 42.5 R cement with limestone powder as a mineral filler. A superplasticizer (SP) and a shrinkage-reducing admixture (SRA) were incorporated to achieve the target water-to-binder ratio. The detailed mix proportions are summarized in Table 1. The mixing procedure followed ASTM C192/C192M [26]. Specimens were demolded after 24 h and subsequently cured in water at 20 °C for 28 days prior to testing.

**Figure 4:**
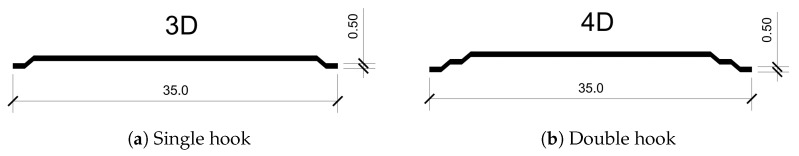
Geometry of the Fe-SMA fibers (units in mm).

The fibers used in this study were produced from Fe-SMA wires with the chemical composition Fe–17Mn–5Si–10Cr–4Ni–1(V,C) (mass%) and pre-strained to 4.0% elongation to store recoverable strain energy within the material [17,27]. The wires were subsequently cut to a total length of 35 mm and their ends shaped using a mechanical punching system to form either a single hook (3D fiber) or a double hook (4D fiber), as illustrated in Figure 4. Both fiber types shared an identical aspect ratio of 70 and a diameter of 0.5 mm.

### 2.2. Specimen Preparation

**Figure 5:**
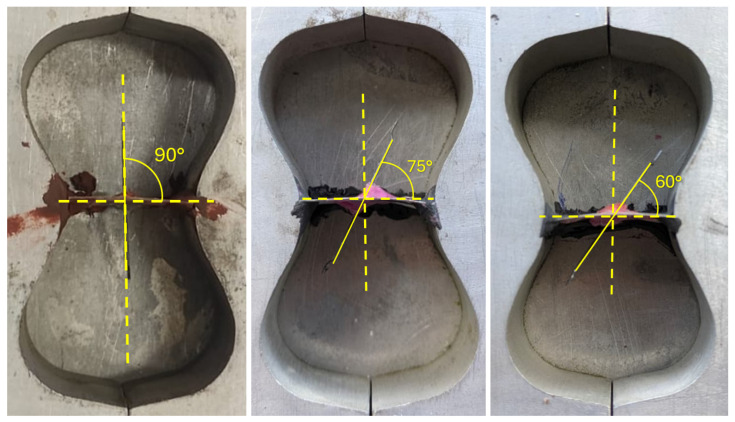
Prepared molds with a 4D Fe-SMA fiber inclined at 90°, 75°, and 60° prior to casting.

Pull-out specimens were prepared using the briquette geometry specified in ASTM C307 [28]. Although this standard was originally developed for direct tensile testing of cementitious materials, its geometry has been widely adopted for single-fiber pull-out experiments [29,30]. A single Fe-SMA fiber was placed at the center of the reduced cross-section prior to casting, with its axis oriented at the desired inclination angle (90°, 75°, or 60° relative to the loading direction), as shown in Figure 5. The fiber inclination was verified using a protractor, while a ruler confirmed the central placement of a thin cardboard interlayer, which was secured at the mid-plane of the mold using clay. Upon demolding, the cardboard divided the specimen into two independent halves, each containing one anchored fiber end with an embedment length of 17.5 mm, ensuring that only the embedded fiber contributed to load transfer during the pull-out test. The fiber was additionally fixed in position using adhesive to prevent any displacement prior to casting. The internal walls and base of each mold were coated with a release agent, applied with care to avoid any contact with the fiber.

**Figure 6:**
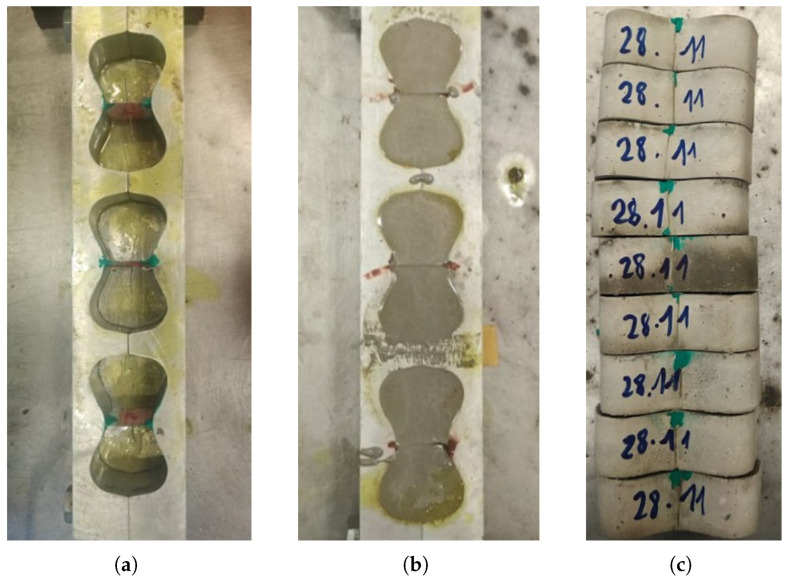
Specimen preparation: (**a**) briquette molds with cardboard interlayers and Fe-SMA fibers secured at the mid-plane prior to casting; (**b**) briquette molds after casting of the HPC mixture; (**c**) demolded briquette specimens following 28-day water curing.

The concrete was mixed following a sequential procedure. First, the fine and coarse sands were dry-mixed for 1 min, after which 20% of the total water was added and mixing continued for a further 1 min, followed by a 1 min rest period to allow the aggregate to absorb moisture. The cement and limestone powder were then introduced and mixed for 2 min. The remaining water, superplasticizer, and shrinkage-reducing admixture were subsequently added, and mixing continued for up to 7–9 min until a homogeneous consistency was achieved. The fresh mixture was then allowed to rest for 1 min to release entrapped air before being poured into the prepared molds. To ensure adequate compaction and eliminate air voids, the molds were vibrated using a rubber hammer for 1 min. The key stages of specimen preparation are illustrated in Figure 6.

Specimens assigned to elevated temperature groups were placed in a laboratory chamber and heated to 100 °C or 200 °C, which was then maintained for 4 h to ensure a uniform temperature distribution throughout the specimen volume; this duration was found to be sufficient to achieve thermal equilibrium even in larger specimens of similar composition [22]. After completion of the heating period, the specimens were removed from the chamber and allowed to cool down to room temperature before testing.

A consistent labeling system was adopted, in which each specimen label consists of the fiber type (**3D** or **4D**), the inclination angle (**90**, **75**, or **60**), and the temperature condition (**A** for ambient, **100** for 100 °C, and **200** for 200 °C). For example, 3D-60-A denotes a briquette with a 3D fiber inclined at 60° tested at ambient temperature, while 4D-75-200 refers to a specimen with a 4D fiber inclined at 75° subjected to thermal treatment at 200 °C. A total of 54 pull-out briquette specimens were prepared (as summarized in Table 2), corresponding to 18 test groups with three replicate specimens per group. This replication level is consistent with the minimum laboratory practice commonly adopted in standardized concrete and mortar testing based on ASTM C192 [26]. Nevertheless, because the number of replicates per group remains limited, the statistical analysis in the present study is reported primarily in a descriptive manner. Unless otherwise stated, the curves shown in Figures 12–15 represent the mean response of the three specimens in each group, while the variability of the maximum pull-out force is reported separately in Figure 16 and Table 3.

In addition to the pull-out briquettes, three types of companion specimens were cast from the same HPC mixture to characterize the mechanical properties of plain concrete under the same thermal conditions. First, nine prismatic specimens in accordance with EN 1015-11 [31], with dimensions of 40 mm × 40 mm × 160 mm, were produced for flexural testing; following the flexural tests, the resulting halves were cut into 40 mm × 40 mm × 40 mm cubes for compressive strength measurements, yielding 18 compression specimens in total. Second, nine plain concrete briquette specimens conforming to ASTM C307 [28]—without any embedded fiber—were prepared to determine the direct tensile strength of the HPC matrix, providing a reference for the fiber–matrix bond characterization. All companion specimens were tested in three groups of three, corresponding to ambient temperature, 100 °C, and 200 °C.

### 2.3. Test Methods

This subsection describes the experimental procedures employed in this study. The pull-out mechanism governing fiber–matrix interaction is first introduced, followed by the thermal treatment protocol applied to the specimens prior to testing. Finally, the mechanical testing procedures for both the pull-out experiments and the companion concrete specimens are presented.

#### 2.3.1. Testing Principle and Measured Quantities

The single-fiber pull-out test was used to quantify the resistance of an embedded Fe-SMA fiber against progressive extraction from the HPC matrix. During testing, the two halves of the ASTM C307-type briquette were pulled apart under displacement control, such that the embedded fiber transferred load across the reduced central section until debonding, hook straightening, and frictional sliding occurred. The applied load and grip displacement were recorded continuously throughout the test. For each test condition, three replicate specimens were tested and the reported load–displacement curves correspond to the mean response of the group. The maximum pull-out force was adopted as the main comparative parameter, while the overall curve shape was used for qualitative interpretation of debonding, mechanical anchorage, and post-peak frictional resistance.

#### 2.3.2. Thermal Treatment

The thermal treatment temperatures of 100 °C and 200 °C were selected to investigate the effect of elevated temperature on the pull-out behavior of Fe-SMA fibers. In Fe-SMA materials, the shape memory effect is thermally activated—upon heating above the austenite start temperature, the material undergoes a martensitic reverse transformation, generating recovery stresses that enhance the fiber–matrix bond. The selected temperature range covers both the onset of this transformation and a higher thermal condition representative of elevated service temperatures, allowing the influence of thermally induced recovery stresses on pull-out resistance to be systematically evaluated [19].

**Figure 7:**
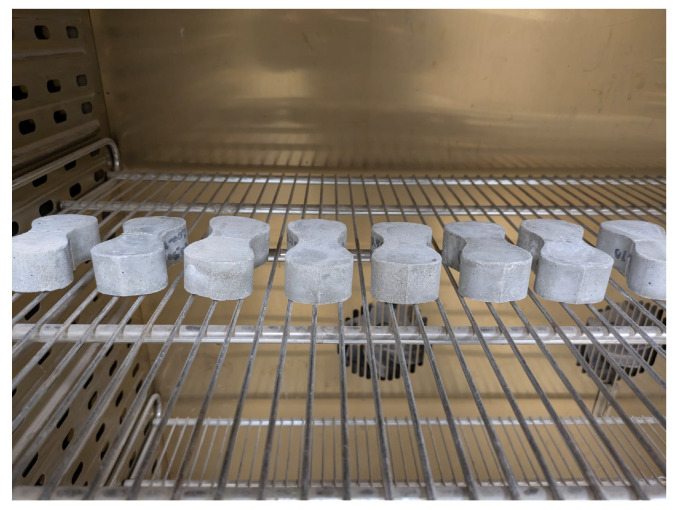
Fe-SMA briquette specimens placed inside the laboratory chamber prior to thermal treatment.

Specimens assigned to elevated temperature groups were placed in a laboratory chamber equipped with internal fans to ensure uniform air circulation and temperature distribution (see Figure 7). The chamber was set to increase the temperature at a rate of 8 °C per minute until the target temperature was reached, which was then maintained for 4 h to ensure a uniform temperature distribution throughout the specimen volume; this heating duration was found to be sufficient to achieve thermal equilibrium in specimens of similar composition and geometry [22]. After completion of the heating period, the specimens were removed from the chamber and allowed to cool down to room temperature before testing.

#### 2.3.3. Mechanical Testing

**Figure 8:**
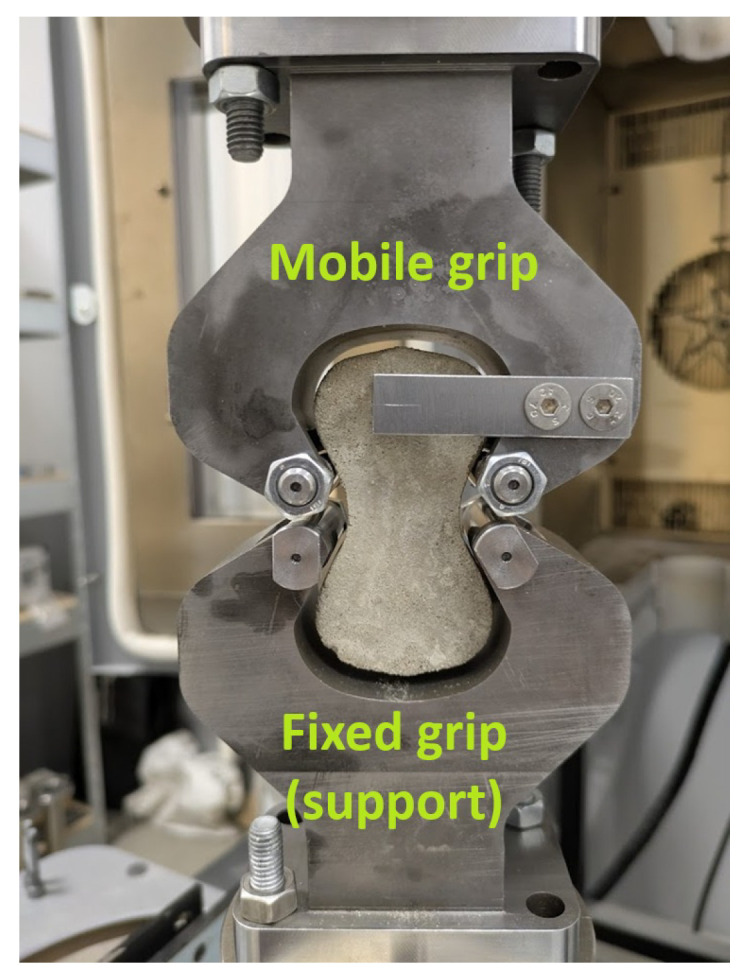
Test setup for single-fiber pull-out testing of Fe-SMA fibers embedded in HPC briquette specimens.

**Figure 9:**
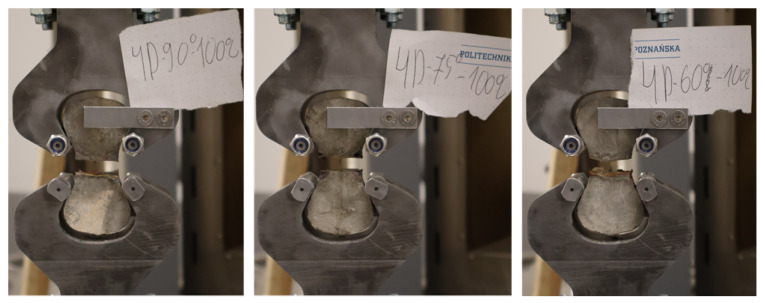
Specimens during pull-out testing at inclination angles of (**left**) 90°, (**center**) 75°, and (**right**) 60°.

Single-fiber pull-out tests were conducted on a displacement-controlled testing machine at a constant loading rate of 0.2 mm/min. Each briquette specimen was mounted in the grip assembly specified in ASTM C307 [28], in which the upper and lower halves of the briquette were held independently by two symmetric metallic grips conforming to the curved geometry of the briquette ends, as shown in Figure 8. As the upper grip moved upward, the two halves were pulled apart, forcing the Fe-SMA fiber to be progressively extracted from the concrete matrix. The pull-out force and grip displacement were recorded continuously throughout the test. Testing was terminated at a grip displacement of 6 mm, at which point all hook deformations had been completed and the fiber response had transitioned to the frictional pull-out regime; beyond this displacement, the force–displacement curve was expected to decrease monotonically with no further significant changes in pull-out resistance. Figure 9 shows representative specimens with 4D fibers at inclination angles of 90°, 75°, and 60° mounted in the grip assembly during testing.

**Figure 10:**
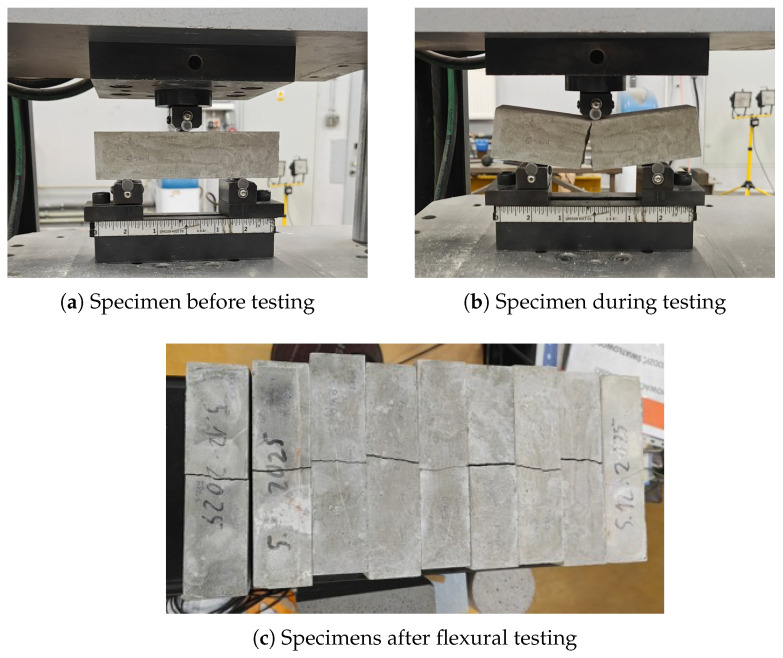
Three-point bending flexural test: (**a**) prismatic 40 mm × 40 mm × 160 mm specimen positioned on rolling supports prior to loading; (**b**) mid-span loading during the test; (**c**) fractured specimens following completion of flexural testing.

The compressive and flexural properties of plain HPC were evaluated on a separate testing machine following EN 1015-11 [31] at a loading rate of 0.2 mm/min. Flexural tests were conducted on 40 mm × 40 mm × 160 mm prismatic specimens subjected to three-point bending, with two rolling supports positioned 100 mm apart and the load applied at mid-span, as shown in Figure 10. Each specimen was loaded to complete failure. Following flexural testing, the resulting halves were cut into 40 mm × 40 mm × 40 mm cubes for compressive strength measurements, with the load applied to the 40 mm × 40 mm cross-section and each cube loaded to complete failure.

**Figure 11:**
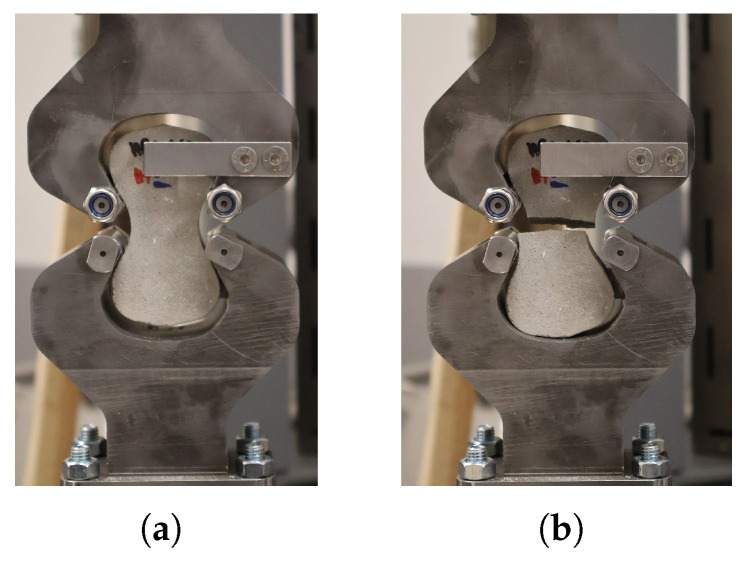
Tensile mechanical test performed on the plain concrete specimen: (**a**) specimen before testing; (**b**) specimen after testing.

The direct tensile strength of plain HPC was determined using plain concrete briquette specimens conforming to ASTM C307 [28], without any embedded fiber, tested under the same grip assembly and loading rate as the pull-out specimens (see Figure 11). Each specimen was loaded to complete failure. These results provide a reference baseline for the fiber–matrix bond characterization, allowing the contribution of the Fe-SMA fiber to be isolated from the intrinsic tensile resistance of the matrix.

## 3. Results and Discussion

**Figure 12:**
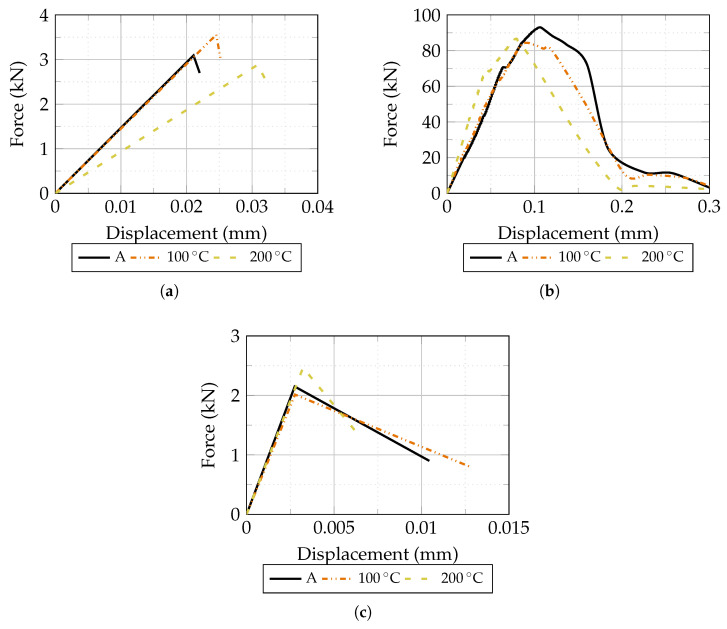
Mean load–displacement response of plain HPC at ambient temperature, 100 °C, and 200 °C: (**a**) flexural test on prismatic specimens; (**b**) compressive test on cubic specimens; (**c**) direct tensile test on briquette specimens.

**Figure 13:**
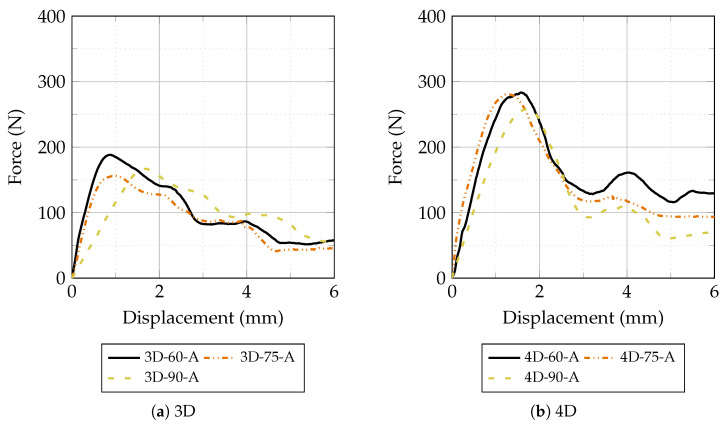
Force–displacement curves of 3D and 4D hooked-end Fe-SMA fibers during pull-out tests at ambient temperature for inclination angles of 60°, 75°, and 90°.

**Figure 14:**
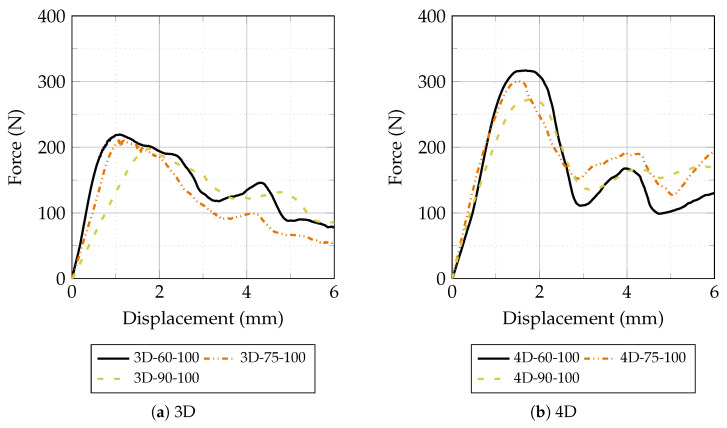
Force–displacement curves of 3D and 4D hooked-end Fe-SMA fibers during pull-out tests at 100 °C for inclination angles of 60°, 75°, and 90°.

**Figure 15:**
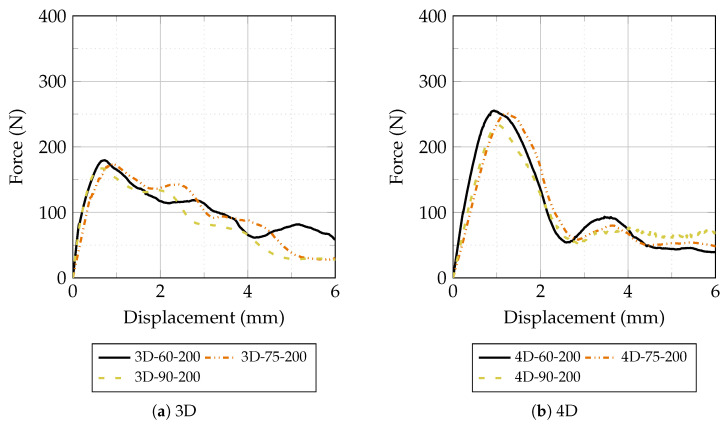
Force–displacement curves of 3D and 4D hooked-end Fe-SMA fibers during pull-out tests at 200 °C for inclination angles of 60°, 75°, and 90°.

**Figure 16:**
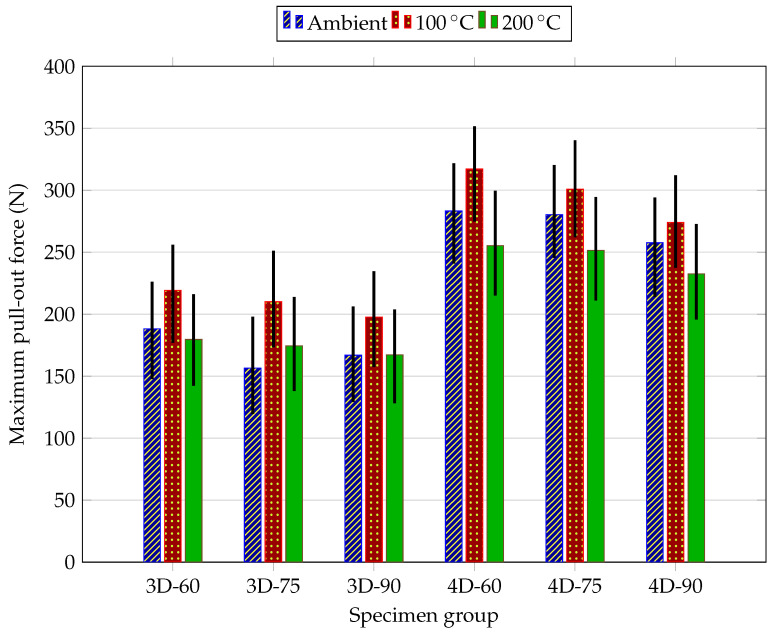
Mean maximum pull-out force for each specimen group at ambient temperature, 100 °C, and 200 °C.

This section presents and discusses the experimental results obtained from the pull-out tests on Fe-SMA fiber briquette specimens and the mechanical characterization of plain HPC. The results are organized as follows: first, the mechanical properties of plain HPC under different thermal conditions are presented; then, the pull-out behavior is analyses with respect to the effects of thermal treatment, fiber geometry, and inclination angle. Unless otherwise stated, Figure 12, Figure 13, Figure 14 and Figure 15 present the mean load–displacement response obtained from three replicate specimens for each test condition. Figure 16 summarizes the mean maximum pull-out force of each group, and the associated variability is quantified by the error bars in Figure 16 and by the descriptive statistics reported in Table 3.

The mean load–displacement responses of plain HPC under flexural, compressive, and direct tensile loading are presented in Figure 12. The HPC matrix demonstrated high mechanical performance under ambient conditions, consistent with the mix design. Exposure to 100 °C produced contrasting effects on the two strength parameters: flexural strength increased by approximately 15%, while compressive strength decreased by approximately 9%. This divergence is consistent with the differing failure mechanisms governing flexural and compressive behavior in cementitious composites: in flexural, initial drying of the matrix at moderate temperatures may promote densification of the microstructure and temporarily enhance bending resistance, whereas compressive failure is more sensitive to thermal degradation of the cement paste even at moderate temperatures [32,33]. At 200 °C, flexural strength decreased relative to ambient but compressive strength recovered slightly compared to 100 °C, reflecting the non-monotonic thermal response of HPC reported in the literature and attributed to competing effects of drying, sintering, and microcracking. The direct tensile strength showed limited sensitivity to temperature up to 100 °C and was slightly higher at 200 °C than at ambient temperature. While unexpected, this result may be related to the redistribution of internal eigenstresses following partial dehydration of the matrix, which can locally increase the apparent tensile capacity of the briquette cross-section. However, given the small sample size (n = 3 per group), this observation should be interpreted with caution and may warrant further investigation. Overall, the results confirm that the HPC matrix retains the majority of its mechanical capacity under the tested thermal conditions, providing a meaningful reference for interpreting the fiber–matrix bond behavior discussed later.

The mean force–displacement curves obtained from pull-out tests at ambient temperature are shown in Figure 13. For both 3D and 4D fiber types, the curves exhibit the characteristic hooked-end pull-out response described in Figure 2, with a well-defined peak force followed by a progressive decrease governed by frictional resistance. At ambient temperature, 4D fibers consistently achieved peak pull-out forces approximately 50–70% higher than 3D fibers across all inclination angles. This substantial advantage confirms that the double-hook geometry provides superior mechanical anchorage compared to the single-hook configuration: the second hook engages an additional bearing zone within the matrix, contributing an independent load-transfer mechanism and producing the characteristic double-peak response visible in the 4D curves [34]. Regarding inclination angle, 60° specimens achieved the highest peak pull-out force for 3D fibers, while for 4D fibers the difference between 60° and 75° was marginal. The 90° specimens consistently exhibited the lowest peak forces for both fiber types, confirming the contribution of the snubbing effect to pull-out resistance: at non-perpendicular inclinations, a transverse force component develops at the fiber exit point, generating additional frictional confinement and increasing the effective pull-out resistance [24,25]. The relatively small difference between 60° and 75° in 4D fibers suggests that the dominant contribution to peak resistance comes from the double-hook anchorage rather than from the snubbing effect at these angles.

Exposure to 100 °C resulted in a notable increase in pull-out resistance across all specimen groups, as shown in Figure 14 and Figure 16: 3D fibers gained approximately 10–17% and 4D fibers approximately 6–12% relative to ambient conditions. This behavior is attributed to the shape memory effect of the Fe-SMA material. Upon heating above the austenite start temperature, the pre-strained fiber undergoes a partial martensitic reverse transformation that generates recovery stresses within the fiber. In a confined composite, these recovery stresses manifest as an increased radial pressure on the surrounding matrix, enhancing frictional resistance along the debonded interface and amplifying the mechanical anchorage at the hook [22]. The greater relative gain observed for 3D fibers compared to 4D fibers may reflect the fact that frictional enhancement plays a larger role in 3D pull-out, since 3D fibers rely more heavily on interface friction once the single hook is straightened. The characteristic double-peak response of 4D fibers is particularly pronounced at 100 °C compared to ambient conditions, consistent with the hypothesis that thermally activated recovery stresses amplify the load transfer at each hook independently. The inclination angle effect remained consistent at 100 °C, with 60° specimens exhibiting the highest peak forces for both fiber types, confirming that the snubbing effect is preserved under thermal activation conditions. The optimal specimen—4D-60-100—achieved the highest peak pull-out force recorded in the entire experimental program, demonstrating the synergistic interaction between double-hook anchorage, moderate fiber inclination, and thermal activation of the shape memory effect.

At 200 °C, pull-out resistance decreased below ambient levels for all specimen groups by approximately 5–12%, as shown in Figure 15. This reduction can be attributed to two concurrent mechanisms acting simultaneously on the fiber–matrix system. First, the degradation of the HPC matrix at elevated temperatures weakens the fiber–matrix interface: thermal exposure promotes dehydration of C-S-H phases, increases capillary porosity, and generates thermal microcracks, all of which reduce the confinement pressure around the fiber and lower both frictional and mechanical anchorage resistance. Second, at temperatures approaching or exceeding the austenite finish temperature of the Fe-SMA alloy, the martensitic reverse transformation may be complete, meaning no additional recovery stresses are generated beyond this point. Furthermore, prolonged exposure to elevated temperatures can partially relax the residual pre-strain stresses stored in the fiber, reducing the driving force for the shape memory effect [22]. Despite this overall reduction, 4D fibers retained a pull-out resistance approximately 40–42% higher than 3D fibers at 200 °C, confirming that the double-hook geometry provides a more robust anchorage mechanism under thermal degradation conditions. The differences between inclination angles became less pronounced at 200 °C compared to lower temperatures, suggesting that matrix damage at the fiber exit point progressively diminishes the effectiveness of the snubbing effect, consistent with findings reported for steel fibers in thermally degraded matrices.

Figure 16 and Table 3 provide an explicit statistical description of the pull-out results by reporting, for each specimen group, the mean maximum pull-out force together with the corresponding minimum–maximum value and percentage standard deviation obtained from the three replicate specimens. The relatively narrow error bars and the low reported scatter, ranging from 3.50% to 9.13%, indicate good repeatability of the experimental results; in experimental mechanics and cement-based materials, coefficients of variation below about 10% are commonly interpreted as slight variation or good repeatability, and the use of mean values together with error bars is a standard way to distinguish general trends from experimental scatter. On this basis, the differences observed between the main groups can be considered meaningful at the descriptive level: the 4D fibers consistently showed higher mean pull-out forces than the corresponding 3D fibers under all thermal conditions, the 100 °C series generally produced the highest mean values, and the 200 °C series showed a reduction relative to 100 °C.

In addition to the general thermal degradation of the bulk matrix, deterioration of the interfacial transition zone (ITZ) should also be considered when interpreting the results at 200 °C. Elevated-temperature exposure can promote dehydration of cement hydration products, increase porosity, and induce microcracking within both the cement paste and the ITZ, thereby reducing the local confinement acting on the embedded fiber. At the same time, the thermomechanical response of Fe-SMA is strongly dependent on activation temperature and restraint conditions; once the reverse martensitic transformation has been substantially completed, further heating may no longer provide additional beneficial recovery stresses, while partial stress relaxation may reduce the effective prestressing contribution of the pre-strained fiber. The observed reduction in pull-out resistance at 200 °C is therefore interpreted as the combined outcome of bond-zone degradation in the surrounding HPC and a less favorable balance between thermal activation and stress retention in the Fe-SMA fiber.

Considering all three experimental variables simultaneously, the results demonstrate clear synergistic effects between fiber geometry, inclination angle, and thermal treatment. The highest pull-out resistance in the entire program was recorded for 4D-60-100, confirming that double-hook geometry, moderate fiber inclination, and partial thermal activation of the shape memory effect act cooperatively to maximize fiber–matrix load transfer. The lowest peak forces were recorded consistently for 3D fibers at 90°, particularly at 200 °C, where the perpendicular single-hook configuration offers limited mechanical anchorage and the snubbing effect is absent. The advantage of 4D over 3D fibers ranged from approximately 50–70% at ambient temperature to approximately 40–42% at 200 °C, indicating that the double-hook geometry is less susceptible to thermal degradation than might be expected. This robustness can be explained by the redundant anchorage mechanism of the second hook: even if the bearing zone of the first hook is partially weakened by matrix degradation, the second hook continues to engage less-affected material further along the embedment length, preserving a significant portion of the total anchorage capacity [34]. These findings have direct implications for the use of Fe-SMA fibers in thermally activated structural applications, where the combined optimization of fiber geometry, orientation, and activation temperature may offer a viable strategy for enhancing crack bridging and tensile performance of HPC composites.

It should be emphasized that the present inclination-controlled pull-out tests isolate the micromechanical role of fiber orientation in a single plane, whereas fibers in real cementitious composites are randomly distributed in three dimensions. Accordingly, the present results should be interpreted as a mechanistic benchmark rather than a direct representation of the full orientation state in structural-scale composites. In practice, the contribution of Fe-SMA fibers to crack bridging will depend not only on the pull-out resistance associated with a given inclination angle, but also on the statistical orientation distribution, local fiber clustering, and crack-plane interception probability. Future work should therefore combine the present single-fiber results with orientation-factor based analyses and composite-scale testing in order to translate the identified micromechanical trends into structural design guidance.

## 4. Conclusions

This study investigated the combined effects of fiber geometry, inclination angle, and thermal treatment on the pull-out resistance of Fe-SMA hooked-end fibers embedded in HPC. A total of 54 briquette specimens were tested across 18 groups, covering two fiber geometries (3D single-hook and 4D double-hook), three inclination angles (60°, 75°, and 90°), and three thermal conditions (ambient, 100 °C, and 200 °C). To the authors’ best knowledge, this is the first study to simultaneously investigate the interaction of hook geometry, fiber inclination angle, and thermal activation on the pull-out resistance of Fe-SMA fibers in HPC. Based on the experimental results, the following conclusions can be drawn:

- The fiber geometry had the most significant influence on pull-out resistance. At ambient temperature, 4D double-hook fibers achieved peak pull-out forces approximately 50–70% higher than 3D single-hook fibers, with this advantage preserved at 200 °C (40–42%). The redundant anchorage provided by the second hook proved robust under thermal degradation, making 4D geometry the preferred configuration for thermally activated Fe-SMA fiber applications.
- Thermal treatment produced a non-monotonic effect on pull-out resistance. Exposure to 100 °C increased resistance by approximately 10–17% for 3D fibers and 6–12% for 4D fibers, attributed to recovery stresses generated by partial martensitic reverse transformation of the pre-strained Fe-SMA material. At 200 °C, resistance decreased below ambient levels by approximately 5–12% for all groups due to concurrent matrix degradation and completion of the martensitic reverse transformation beyond the austenite finish temperature. Within the investigated temperature range, exposure to 100° produced the highest pull-out resistance, whereas exposure to 200° reduced the pull-out resistance below the ambient-temperature level.
- The inclination angle consistently influenced pull-out resistance across all thermal conditions. Specimens at 60° achieved the highest peak forces and perpendicular (90°) specimens the lowest, confirming the contribution of the snubbing effect. The angular sensitivity diminished at 200 °C, indicating that thermal matrix degradation reduces the effectiveness of the snubbing mechanism.
- The synergistic interaction between all three variables was confirmed by the 4D-60-100 configuration achieving the highest pull-out resistance in the entire experimental program. This result demonstrates that combined optimization of fiber geometry, inclination angle, and thermal activation temperature offers a viable strategy for maximizing fiber–matrix load transfer in thermally activated Fe-SMA fiber-reinforced HPC composites.

The present study has several limitations that should be acknowledged. The cardboard interlayer used to control fiber inclination was relatively thin and deformable, meaning that actual angles may have deviated slightly from the intended values. Furthermore, the inclination angle was controlled in a single plane only, whereas in practice fibers are randomly oriented in three dimensions. The number of replicates was limited to three specimens per group, which constrains the statistical significance of the results, and only three inclination angles were investigated, providing limited resolution of the angular dependence. Finally, the casting technique may have introduced geometric variability in fiber positioning between specimens.

Future research should address these limitations by developing an improved specimen preparation system for precise three-dimensional control of fiber inclination. Additional inclination angles and higher thermal treatment temperatures should be investigated to fully characterize the angular and thermal dependence of pull-out resistance. Studies involving larger fiber populations per specimen would allow the transition from single-fiber pull-out to composite behavior to be examined. Finally, the development of a validated numerical model incorporating hook geometry, fiber inclination, and thermally activated recovery stresses would provide a powerful tool for parametric studies and structural design optimization.

## Figures and Tables

**Table 1:** High performance concrete mixture proportions per 1 m^3^.

Constituent	Mass (kg) in m^3^
Portland cement CEM II 42.5 R	620
Limestone powder	480
Fine sand 0–0.5 mm	700
Coarse sand 0.5–1.0 mm	300
Water	190
Superplasticizer	16.5
Shrinkage-reducing admixture	16.5

**Table 2:** Test matrix for the pull-out briquette specimens.

Fiber Type	Angle	Temperature	Number of Briquettes
3D	90°	Ambient	3
		100 °C	3
		200 °C	3
	75°	Ambient	3
		100 °C	3
		200 °C	3
	60°	Ambient	3
		100 °C	3
		200 °C	3
4D	90°	Ambient	3
		100 °C	3
		200 °C	3
	75°	Ambient	3
		100 °C	3
		200 °C	3
	60°	Ambient	3
		100 °C	3
		200 °C	3
Total number of briquettes			54

**Table 3:** Descriptive statistics of the maximum pull-out force for each specimen group (units for Mean and Max/Min are in N).

	Ambient			100 °C			200 °C		
Specimen	Mean	Max/Min	SD (%)	Mean	Max/Min	SD (%)	Mean	Max/Min	SD (%)
3D-60	188.20	192.71/181.16	5.98	219.28	222.63/210.38	6.75	179.59	182.64/175.71	3.50
3D-75	156.52	164.57/152.97	6.27	210.03	217.80/206.94	5.86	174.46	180.32/171.62	4.54
3D-90	166.91	172.84/162.72	5.14	197.66	201.25/190.85	5.38	167.28	170.25/161.73	4.53
4D-60	283.23	288.28/274.39	7.17	317.07	318.17/308.24	6.30	255.24	266.16/248.49	9.13
4D-75	280.37	286.83/279.00	4.70	300.80	306.71/295.49	5.62	251.45	261.00/244.61	8.29
4D-90	257.62	260.55/247.66	7.36	274.05	278.66/270.88	3.98	232.58	239.26/229.29	5.25

## Data Availability

The original contributions presented in this study are included in the article. Further inquiries can be directed to the corresponding author.
